# Early effects of *Staphylococcus aureus* biofilm secreted products on inflammatory responses of human epithelial keratinocytes

**DOI:** 10.1186/1476-9255-11-17

**Published:** 2014-06-03

**Authors:** Amy Tankersley, Mark Barton Frank, Melissa Bebak, Robert Brennan

**Affiliations:** 1 Biology Department, University of Central Oklahoma, 100 North University Drive, Edmond, Oklahoma 73034, USA; 2 Microarray Research Facility, Arthritis and Clinical Immunology Program, Oklahoma Medical Research Foundation, Oklahoma City, Oklahoma, USA

**Keywords:** *Staphylococcus aureus*, Biofilms, Keratinocytes, Inflammation, Nitric oxide, Gene expression microarray

## Abstract

**Background:**

Chronic wounds such as diabetic foot ulcers, pressure ulcers, and venous leg ulcers contribute to a considerable amount of mortality in the U.S. annually. The inability of these wounds to heal has now been associated with the presence of microbial biofilms. The aim of this study was to determine if products secreted by *S. aureus* biofilms play an active role in chronic wounds by promoting inflammation, which is a hallmark of chronic wounds.

**Methods:**

*In vitro* experiments were conducted to examine changes in gene expression profiles and inflammatory response of human epithelial keratinocytes (HEKa) exposed to products secreted by *S. aureus* grown in biofilms or products secreted by *S. aureus* grown planktonically.

**Results:**

After only two hours of exposure, gene expression microarray data showed marked differences in inflammatory, apoptotic, and nitric oxide responses between HEKa cells exposed to *S. aureus* biofilm conditioned media (BCM) and HEKa cells exposed to *S. aureus* planktonic conditioned media (PCM). As early as 4 hours post exposure, ELISA results showed significant increases in IL-6, IL-8, TNFα, and CXCL2 production by HEKa cells exposed to BCM compared to HEKa cells exposed to PCM or controls. Nitric oxide assay data also showed significant increases in nitric oxide production by HEKa cells treated with BCM compared to HEKa cells treated with PCM, or controls.

**Conclusions:**

Taken together, these results support and extend previous findings that indicate products secreted by *S. aureus* biofilms directly contribute to the chronic inflammation associated with chronic wounds.

## Background

Each year millions of people are stricken with chronic wounds such as diabetic foot ulcers, pressure ulcers, and venous leg ulcers that contribute to the morbidity and mortality in the U.S. annually [1-3]. Recent evidence indicates that bacterial biofilms are present in chronic wounds more often than in acute wounds and are related to the wounds inability to heal [4-6]. Bacterial biofilms consist of bacterial communities embedded in a self-made extracellular polysaccharide matrix that are often resistant to a variety of antibiotics [7]. Chronic wounds provide an excellent environment for this matrix due to the tissue surface for growth, poor blood flow, and poor oxygen flow that inhibit host cell defenses against the bacteria [8]. The surface of chronic wounds allow bacteria to readily adhere to a surface and relinquish their planktonic state to form an aggregate of bacteria that enable the newly formed biofilms to better survive in their environment [9]. The inhibitions of the host cell defenses allow the bacteria to cooperate in the distribution of nutrients, removal of wastes, and to resist host defense mechanisms [6].

The establishment of *S. aureus* biofilms in chronic wounds relies on the bacterial community’s ability to adhere to tissue. After attachment, the bacteria rapidly secrete cell signaling molecules that coordinate activities of the bacteria through a process known as quorum sensing [10]. Biofilm formation by *Staphylococcus aureus* has been shown to be highly dependent on the staphylococcal accessory regulator (*sarA)*[11] and to a certain degree on the (*icaABCD)* operon [12-15], and the *walRK* operon [16]. Mutations in either *sarA* or the *ica* operon have been associated with reduced capacity of *S. aureus* to form biofilms. There is significant evidence that the molecules in the matrix of biofilms lead to impermeability to antibiotics and host defenses [17]. While the idea that biofilms affect wound healing has been established, the exact role of biofilms in chronic wounds is not well understood [7]. Biofilm’s resistance to antibiotics make the infections difficult to resolve and more likely to become chronic or recurrent infections in part due to the inhibition of the re-epithelialization process [18].

Wound healing is a complex series of pathogen and host cell interactions. Most chronic skin wound healing is mediated by keratinocytes [19,20]. The ability of keratinocytes to migrate to the site of the wound and respond to inflammatory signals to eliminate infection and complete the process of reepithelialization is key to the ability of chronic wounds to heal [20]. Keratinocytes have been shown to express toll-like receptors 1–6 and 9, which can allow them to act as a first responder against pathogenic microorganisms. For example, *S. aureus* can activate nuclear transcription factor kB (NF-κB) in keratinocytes. Activated NF-κB then translocates into the nucleus and induces the transcription of NF-κB controlled genes such as interleukin 8 (IL-8) and nitric oxide synthase (iNOS) [21,22]. The role that keratinocytes play in wound pathogenesis makes them an excellent model to investigate the pathogenesis of wound healing *in vitro*.

Inflammatory response of the host is important to the ability of wounds to heal. Biofilms have been shown to contribute to the failure of wounds to reepithelialize through the activation of the β-catenin/c-myc pathways which is in part attributed to the unresponsiveness of cells at the wounds edge [23-25]. A part of that inflammatory response is the production of inflammatory cytokines that serve to mediate host immune responses. Recent evidence has revealed that *S. aureus* biofilms affect the gene regulation and cytokine production of keratinocytes and thus may affect the way that wounds heal [26]. The goal of this project was to further investigate and compare the effects of S*. aureus* biofilm secreted products and *S. aureus* planktonic secreted products on gene expression profiles and inflammatory responses of human epithelial keratinocytes (HEKa). The hypothesis is that products secreted by *S. aureus* growing as a biofilm actively impair wound healing by promoting inflammatory responses in keratinocytes. To test this hypothesis, we investigated and compared inflammatory responses of HEKa cells exposed to BCM and PCM at both transcriptional and translational levels.

## Methods

### Preparation of biofilm conditioned media

An overnight culture of *Staphylococcus aureus* ATCC 6538 in 5.0 ml of tryptic soy broth (TSB) was incubated statically at 37°C for 24 hours. Tissue culture inserts were placed in a 24 well plate and inoculated with 10 μl of overnight culture and 500 μl of TSB and inoculated at 37°C for 72 hours. Every 24 hours during that 72 hour period the TSB supernatant was removed, the inserts were moved to new wells in the 24 well plates, and 500 μl of fresh TSB was added to the wells. At the end of 72 hour period the TSB was removed and 500 μl of phosphate buffered saline pH 7.4 (PBS) was added and left for 1 hour to wash the remaining TSB from the tissue culture insert. After the removal of the PBS, 500 μl of Epilife media (Invitrogen, Carlsbad, CA) was added and incubated for 24 hours at 37°C. The new biofilm conditioned media was then removed from the well and filtered with a 0.45 μm syringe and collected in 15 ml centrifuge tubes. This BCM collecting and filtering procedure was repeated every 24 hours for 3 days. The collected BCM was then pooled and frozen at -20°C until it was needed.

### Preparation of planktonic conditioned media

An overnight culture was created by inoculating a colony of *S. aureus* ATCC 6538 into 5.0 μl of TSB for 24 hours at 37°C on a rotary shaker set at 150 rpm. After incubation the *S. aureus* culture was centrifuged for 7 minutes at 1500 rpm. The supernatant was then replaced with PBS, and the pellet was re-suspended by thoroughly mixing with a pipette. The *S. aureus* was then centrifuged for 7 minutes at 1500 rpm and the PBS was decanted. Five milliliters of Epilife media with human keratinocyte growth supplement was then added to the washed *S. aureus* culture and mixed thoroughly with a pipette. The *S. aureus* in the Epilife media was then incubated for 24 hours at 37°C on a rotary shaker set at 150 rpm. After 24 hours the culture was centrifuged at 1500 rpm for 7 minutes and the supernatant was decanted and filtered with a 0.45 μm syringe and stored at -20°C until needed.

### Culturing of human keratinocytes

HEKa cells (Invitrogen, Carlsbad, CA) were seeded into a T-25 and T-75 tissue culture flasks at 1 x 10^4^ cells/ml along with Epilife media with human keratinocyte growth supplement and 100μg/ml and 100 U/ml of pen/strep and incubated at 37°C in a CO_2_ incubator. Every 48 hours the Epilife media was changed until the cells reached 80-90% confluence. In our hands, HEKa cells would start to become senescent at about passage 8 or 9. To prevent cell senescent from altering the results all experiments were performed with cells at passages 3 or 4.

### XTT HEKa cell viability assay

BCM and PCM that was created previously were warmed to room temperature and diluted to1 mg/ml in Epilife media with human keratinocyte growth supplement. HEKa cells were cultured until they were at 80-90% confluence and then the original Epilife media with human keratinocyte growth supplement media was removed and 300 μl of either the PCM, BCM or media was added to the wells in triplicate and incubated for 0, 2, 4, 8, 24, and 48 hours. At the designated time 60 μl of activated 2, 3-bis-(2-methoxy-4-nitro-5-sulfophenyl)-2H-tetrazolium-5-carboxanilide (XTT) solution was added and absorbance read at 450 nm.

### Genomic responses, statistics and bioinformatics analyses

HEKa cell genomic responses to PCM, BCM and Epilife media were also evaluated. HEKa cells were cultured and plated in a 24 well plate as described above and were grown to 80-90% confluence. After reaching 80-90% confluence the cells were exposed to 1.0 ml of PCM, BCM or Epilife media for a period of 0 and 2 hours. At the designated times points the BCM, PCM, and Epilife media was removed, and the cells liberated from the wells for RNA extraction using Qiagen RNAeasy Cell Protect kit (Qiagen Inc. Valencia, CA). The concentrations of the RNA were determined using a Nanodrop spectrophotometer. RNA was diluted to a 0.1 μg/ml concentration in RNase-free water and transported to Oklahoma Medical Research Foundation (OMRF) for microarray analysis. RNA quality was assessed using capillary gel electrophoresis (2100 Bioanalyzer, Agilent Technologies, Santa Clara, CA). Samples were labeled using Illumina TotalPrep RNA Amplification kit (Ambion/Life Technologies, Carlsbad, CA) and hybridized to whole genome gene expression microarrays (Illumina Human HT-12 v4 BeadChips, Illumina Inc., San Diego, CA) according to manufacturers’ instructions. Microarrays were stained and washed under high stringency conditions and were then scanned on an Illumina iSCAN scanner. Signal intensity values were obtained using GenomeStudio software (Illumina, v2011.1) and quantile normalized and log transformed using MatLab software (Mathworks, Inc., Natick MA) prior to importing into BRB-Array Tools ( National Cancer Institute, Biometric Research Branch, Rockville, MD). Genes were then filtered using the Log Expression Variance Filter to screen out genes that are not likely to be informative based on the variance of each gene across the arrays. Biological replicates for each group were designated and statistically significant differentially expressed genes were identified using a 5% false-discovery rate and a minimum 1.5 fold change between the BCM and the PCM samples [27]. Bioinformatics analyses of differentially expressed genes were performed using Ingenuity Pathways Analysis software (Ingenuity Systems, Redwood City, CA).

### Detection of inflammatory cytokines

HEKa cells were cultured to 80-90% confluence in 24 well plates 1.0 ml of BCM, PCM or Epilife media without pen/strep was added in triplicate cultures for periods of 0, 2, 4, and hours. At the designated time points the BCM, PCM and Epilife media was collected and stored in 1.5 ml microcentrifuge tubes at -20°C until ELISA tests were performed. The concentrations of cytokines in the PCM, BCM, and Epilife media products that were exposed to HEKa cells were quantified and analyzed. Quantification of TNF-α IL-1, IL-6, and IL-8 was performed using the corresponding Quantikine Colorimetric sandwich ELISA assay kit (R&D Systems, Minneapolis, MN) following the manufacturer’s instructions. CXCL2 was quantified using Abnova Enzyme-Linked Immunoabsorbant Sandwich Elisa Assay Kit (Abnova Industries, Taipei City, Taiwan).

### Ntiric oxide detection

Cell culture supernatants were collected, and the accumulation of nitrite (NO_2_^-^), a stable end product of NO formation, was measured as an indicator of NO production. The concentration of nitrite in the samples was calculated from a standard curve of sodium nitrite [28]**]**. Briefly, at four and eight hours post exposure one hundred microliters of supernatants were collected from HEKa cells exposed to PCM, BCM, or control media samples and added in triplicate to the 96 well plates. One hundred microliters of Griess reagent was then added to all wells and incubated at room temperature for 15 minutes, and then absorbance was measured at 570 nm. After incubation the optical densities of each well were read on a Thermo Scientific Multiskan MCC microplate reader (Fisher Scientific, Pittsburgh PA) at 570 nm.

### Statistical analysis

To determine if there were significant differences in the cell viability of HEKa cells exposed to PCM, BCM and the control media two-tailed t-tests were completed. To determine if there were significant differences in the mean concentration of the cytokines or nitric oxide between the three types of materials, two-tailed t-tests were completed along with ANOVA and Tukey’s statistical methods. An ANOVA resulting in a significant p-value (*p* < 0.05) was followed by Tukey’s Honestly Significant Difference to determine specifically which means were significantly different.

## Results

### HEKa cell viability

**Figure 1:**
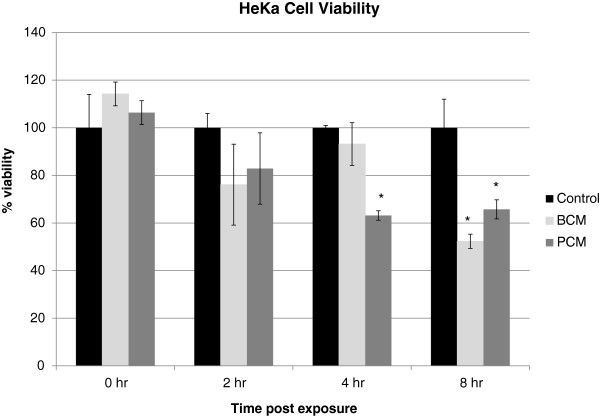
**HEKa cell viability.** HEKa cells were treated with Media, PCM or BCM for up to 8 hours. At 0, 2, 4, and 8 hours post exposure XTT was added and the absorbance read at 540nm. Results represent the mean and standard deviation of three independent experiments. Percent viabilities are stated as percent viable of untreated controls at each time point. (*) indicates a statistically significant difference between the treatment and the control (p < 0.05).

HEKa cell viability experiments were conducted to determine at which point in time if any products secreted by *S. aureus* growing as a biofilm or *S. aureus* growing planktonically significantly reduced viability of HEKa cells. The XTT viability assay was used to determine the loss of HEKa viability after exposure to PCM, BCM, or Epilife media. After 8 hours exposure there was a significant reduction of keratinocyte viability in PCM and BCM exposed cells (Figure 1). Based on these results it was established that sampling time points of 2 hours for microarray analysis and 4 and 8 hours for inflammation analysis would be used.

### Transcriptional responses of HEKa cells exposed to BCM or PCM

**Figure 2:**
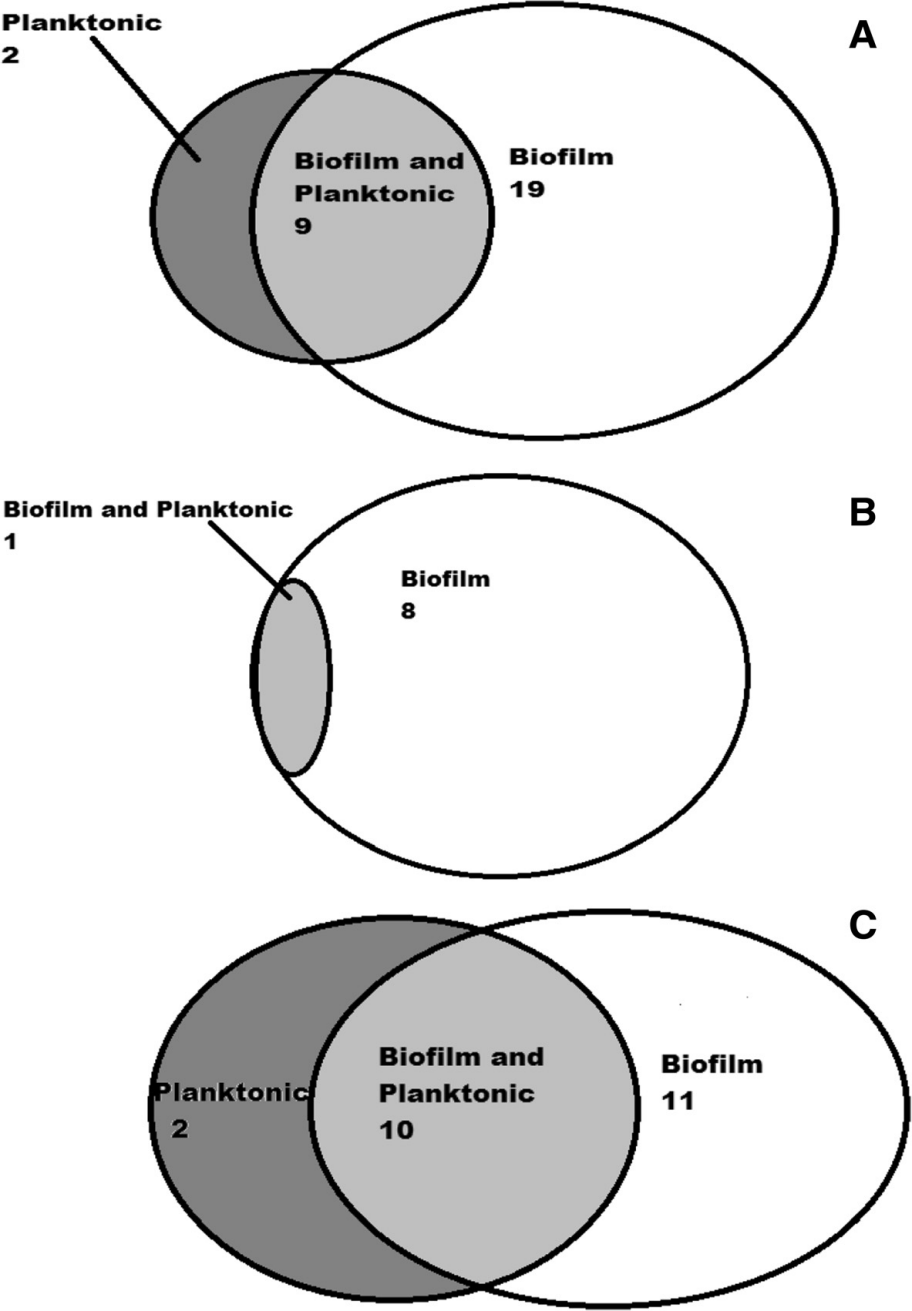
**Venn diagrams representing the number of genes that were altered in HEKa cells exposed to BCM (on the right), PCM (on the left), or shared genes (in the middle).** The Venn diagram **(A)** represents genes associated with inflammation, **(B)** represents genes associated with NO production, and **(C)** represents genes associated with apoptosis.

We predicted that HEKa cells exposed to *S. aureus* biofilm secreted products would display differential genes expression when compared to HEKa cells exposed to *S. aureus* planktonically secreted products or the media controls. To test this hypothesis we extracted HEKa cellular RNA after 2 hours of exposure to BCM, PCM, or media controls and subjected the RNA to Illumina microarray analysis in order to evaluate differential gene expression. The data was filtered so that only genes that had a 1.5 fold changes or greater over the media control were shown (Table 1). Analysis revealed that HEKa cells exposed to BCM or to PCM had genes associated with inflammation, apoptosis, and nitric oxide production upregulated when compared to the media controls. When cells exposed to BCM were compared to those cells exposed to PCM, the cells exposed to BCM had an increase in the number and fold increases of transcriptional products. The microarray analysis identified 43 genes that were uniquely expressed in BCM exposed HEKa cells with a 1.5 or greater fold change in expression that were not expressed in HEKa cells exposed to PCM or the media control (Table 1). Of these genes 42 had an upregulation of expression over the control. Of these upregulated genes, 19 are associated with inflammatory response in eukaryotic cells. Eleven genes associated with inflammation were also differentially expressed in HEKa cells exposed to PCM, 9 of which were also differentially expressed in cells exposed to BCM (Table 2) and (Figure 2A). The largest increase in gene expression in HEKa cells exposed to BCM for genes associated with inflammatory response versus HEKa cells exposed to BCM were DUSP1, CXCL2, IL-8, ATF3, IL-6 and NFKBIA (Table 1 ).

**Table 1:** Change in gene expression profiles for genes that were uniquely expressed in BCM exposed HEKa cells after 2 hr of exposure

**Gene symbol (function)**	**Fold-change BCM v Cont**	**Fold-change PCM v Cont**
CXCL2 (inflammation)	15.082	-
IL8 (inflammation)	9.530	-
DUSP1 (inflammation/NO prod.)	9.061	-
IL6 (inflammation/NO prod.)	4.623	-
NFKBIA (inflammation)	4.093	-
EFNA1 (inflammation)	3.990	-
TNFAIP3 (inflammation)	3.964	-
ADM (inflammation/NO prod.)	2.917	-
CXCL1 (inflammation)	2.850	-
IL1B (inflammation/NO prod.)	2.586	-
ZFP36 (inflammation)	2.540	-
IFI27 (inflammation)	2.411	-
TNF (inflammation/NO prod.)	2.333	-
IL1A (inflammation/NO prod.)	2.113	-
PTGS2 (inflammation/NO prod.)	2.084	-
SMAD7 (inflammation)	2.073	-
IL20 (inflammation)	1.655	-
IL24 (inflammation)	1.540	-
MMP1 (inflammation)	-1.650	-
FOS (apoptosis)	8.433	-
ZC3H12A (apoptosis)	4.361	-
NR4A2 (apoptosis)	4.121	-
SGK1 (apoptosis)	3.045	-
CYP1B1 (apoptosis)	2.709	-
CYR61 (apoptosis)	2.587	-
BMP2 (apoptosis)	2.481	-
SLC25A24 (apoptosis)	2.467	-
CEBPD (apoptosis)	2.419	-
IFI27 (apoptosis)	2.411	-
BIRC3 (apoptosis)	1.643	-
HES1 (growth & proliferation)	4.280	-
DUSP10_v3 (growth & proliferation)	4.091	-
EFNA1 (growth & proliferation)	3.990	-
PPP1R15A (growth & proliferation)	3.582	-
JUN (growth & proliferation/NO prod.)	3.523	-
PPP1R10 (growth & proliferation)	3.099	-
HBEGF (growth & proliferation)	3.053	-
CYR61 (growth & proliferation)	2.587	-
CEBPD (growth & proliferation)	2.419	-
JUNB (growth & proliferation)	2.405	-
SMAD7 (growth & proliferation)	2.073	-
DUSP10_v1 (growth & proliferation)	1.764	-
CYR61 (wound healing)	2.587	-

Genes identified through Illumina microarray results filtered to represent a 1.5 or greater fold change over the expression in the control sample.

**Table 2:** Change in gene expression profiles for genes associated with Inflammation

**Gene symbol**	**Fold-change BCM v Cont**	**Fold-change PCM v Cont**
ATF3	8.342	1.663
FOXA2	3.139	2.715
BCL6	2.801	1.515
SERPINB13	2.513	2.768
RHOB	2.443	1.464
MMP28	2.117	1.672
IL1F9	1.662	2.438
PBK	-2.057	-1.882
TK1	-2.168	-2.029
CXCL2	15.082	-
IL8	9.530	-
DUSP1	9.061	-
IL6	4.623	-
NFKBIA	4.093	-
EFNA1	3.990	-
TNFAIP3	3.964	-
ADM	2.917	-
CXCL1	2.850	-
IL1B	2.586	-
ZFP36	2.540	-
IFI27	2.411	-
TNF	2.333	-
IL1A	2.113	-
PTGS2	2.084	-
SMAD7	2.073	-
IL20	1.655	-
IL24	1.540	-
MMP1	-1.650	-
IL28RA	-	1.589
CXCR7	-	2.435

Genes identified through Illumina microarray results filtered to represent a 1.5 or greater fold change over the expression in the control sample.

Nitric oxide (NO) is an important molecule for inflammation and the reepithelialization of skin. Low concentrations of NO have been found to inhibit adhesion molecule suppression cytokines and chemokine synthesis and leukocyte adhesion and migration [29,30]. This promotes collagen production either directly or through mediators and thus affects collagen synthesis or breakdown in the wound [31]. While low amounts of NO can be beneficial to wound repair large amounts of NO generation has been shown to be toxic, pro-inflammatory, and cause the wound to enter a cycle where healing does not occur [32,33]. Because of this, microarray results were filtered to look for genes that are associated with the production of nitric oxide (NO). The results showed that at two hours after treatment there were several differentially expressed genes associated with NO production that were uniquely upregulated in HEKa cells exposed to BCM (Table 1). This upregulation was not seen in HEKa cells treated with PCM for two hours. Of these genes DUSP1, JUN, IL-6, and ADM were up regulated the most. Most of the genes above are linked to the production of IFN-γ and TNF-α. Both IFN-γ and TNF-α have been linked to an increase of NO in human keratinocytes [34]. Oxidative stress has been found to increase the amount of IFN-γ produced by the cells which in turn regulates the transcription of DUSP1 and ADM which ultimately leads to increases in apoptosis. IFN-γ along with TNF-α stimulates the production of nitric oxide in keratinocytes as a response to oxidative stress [35]. IFN-γ has been found to increase the production of NO in keratinocytes as much as 20-30%. IFN-γ has also been linked to an increase in production of IL-6 along with NO production [36]. In addition to upregulation of genes associated with NO production, we observed down-regulation of ARG1, which is known to be associated with NO production in both BCM and PCM, exposed HEKa cells relative to control media (Table 3) and (Figure 2B). Overproduction of ARG1 has been linked to keratinocytes overproducing NO in patients with psoriasis and basal carcinomas. The inhibition of ARG1 may then allow NO to inhibit cell proliferation and the failure of wounds to reepithelialize [37].

**Table 3:** Gene expression profiles for genes associated with NO production

**Gene symbol**	**Fold-change BCM v Cont**	**Fold-change PCM v Cont**
ARG1	-3.042	-2.499
DUSP1	9.061	-
IL6	4.623	-
JUN	3.523	-
ADM	2.917	-
IL1B	2.586	-
TNF	2.333	-
IL1A	2.113	-
PTGS2	2.084	-

Genes identified through Illumina microarray results filtered to represent a 1.5 or greater fold change over the expression in the control sample.

### Inflammatory cytokine responses in HEKa cells exposed to BCM or PCM

**Figure 3:**
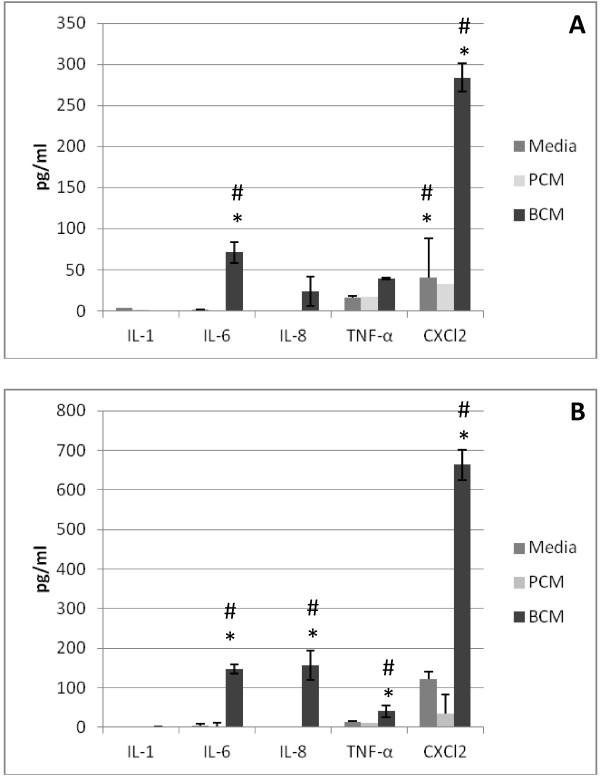
**ELISA results for PCM, BCM and control treated HEKa cells.** HEKa cells were treated with Media, PCM or BCM for up to 8 hours. The levels of cytokine production were measured after 4 and 8 hours of exposure. **(A)** Cytokine measurements after 4 hours of exposure. **(B)** Cytokine measurement after 8 hours of exposure. Measurements are reported in pg/ml concentrations. Analysis of variance (ANOVA) and Tukey’s Honestly Significant Difference were performed to identify statistically significant differences. Results represent the mean and standard deviation of three independent experiments. (#) indicates a significant difference between PCM and BCM and (*) indicates a statistically significant difference between BCM and media (p < 0.05).

In an effort to corroborate the microarray results, we assessed the production of the protein levels of several of the most markedly upregulated genes associated with an inflammatory response. ELISAs were used to measure the production of IL-1, IL-6, IL-8, TNF-α, and CXCL2. Cytokine measurements were performed at 4 and 8 hours post exposure to BCM or PCM. ELISA data showed that as soon as 4 hours post exposure, there were significantly greater (p < 0.05) levels of IL-6, TNF-α, and CXCL2 in HEKa cells exposed to BCM compared to HEKa cells exposed to PCM or control media (Figure 3A). The levels of IL-6 and CXCL2 were even greater at 8 hours post exposure and the significant differences between the groups were maintained (Figure 3B). In addition to these three cytokines, the levels of IL-8 were also significantly greater (p < 0.05) after 8 hours of exposure in HEKa cells exposed to BCM compared to HEKa cells exposed to PCM, or controls (Figure 3B). PCM exposed HEKa cells did not produce significantly greater cytokine levels compared to the media controls at either time point.

### NO responses in HEKa cells exposed to BCM or PCM

**Figure 4:**
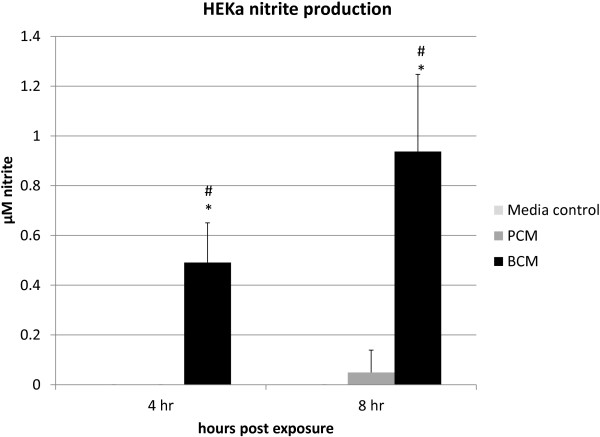
**Nitrite assay.** HEKa cell culture supernatants were collected and treated with Griess Reagent and absorbance was measured at 570nm. Measurements were taken at 4 and 8 hours. Measurements are reported in μM. ANOVA and Tukey’s Honestly Significant Difference were performed to identify statistically significant differences. Results represent the mean and standard deviation of three independent experiments. (#) indicates a statistically significant difference between the PCM and BCM and (*) indicates a statistically significant difference between media and BCM (p < 0.05).

The microarray data revealed an increase in the expression of genes associated with the production of nitrite in BCM-exposed HEKa cells. In an effort to corroborate these results, we performed nitric oxide assays on the HEKa cell culture supernatants to quantify the amount of nitrate produced by the HEKa cells exposed to PCM, BCM and control media. The nitrite assay data showed that HEKa cells exposed to BCM produced significantly greater (p < 0.05) amounts of nitrite at both 4 and 8 hours post exposure than HEKa cells exposed to PCM or the media controls (Figure 4). There were no detectable levels of nitrite from the media controls.

## Discussion

The data collected in this study reveal a clearer picture of the role that *S. aureus* biofilms play on cultured keratinocytes. Keratinocytes serve as the primary cell type in the epidermis and primarily function in providing a barrier between the external and internal environment. When breaks in this barrier occur, basal keratinocytes migrate to the site and reepithelization ensues. Chronic wounds are characterized by prolonged inflammation and the failure of wound reepithelization [8]. Chronic wounds activate a number of inflammatory pathways that lead to the prevention of keratinocyte migration, growth, and differentiation and thus failure of wounds to heal [25]. Due to their importance in chronic wound pathogenesis HEKa cells were chosen for experiments on the effect of biofilms on early chronic wound pathogenesis. Kirker et al. [8] demonstrated that the viability of HEKa cells was significantly reduced when exposed to BCM or PCM for 24 hours. Results from our HEKa cell viability assays showed that viability was significantly reduced by BCM or PCM in as little as 8 hours of exposure (Figure 1), indicating that our findings are in general agreement with previous findings of Kirker et al. [8]. Visual inspection of the cells showed morphological differences between HEKa cells exposed to the two treatment conditions. Evidence of cellular stress in the form of rounding of cell membranes and decreased culture confluency were observed in HEKa cells exposed to BCM after 8 hours of exposure that was not seen in HEKa cells exposed to PCM or the control media (data not shown). Based on the viability assay results and these morphological changes, transcriptional changes were measured at two hours after treatment to detect early transcriptional changes in cell populations with high viabilities. For inflammatory cytokine response and NO production 4 and 8 hour time intervals were used. These time points were selected in an effort to measure downstream effects of transcription and inflammatory responses.

Increased apoptotic effects in HEKa cells exposed to *S. aureus* biofilm secreted products provides greater understanding of the pathogenesis of wound healing. Decreased cell viability and the inability of wounds to heal have been linked to chronic wounds associated with biofilms [8]. Figure 2C shows alterations in transcription of genes associated with apoptosis in BCM- and PCM-exposed HEKa cells. This correlates with our cell viability data (Figure 1) which reveals a statistically significant loss of HEKa cell viability in BCM and PCM after 8 hours. There are several definitions of what an actual chronic wound is, but some common themes are a prolonged inflammatory phase to the wounds and the failure of the wound to respond to standard treatments [38]. These wounds fail to reepithelialize which is due in part to the decrease of cell production and the increase in cell death. Our data indicate that there is an increase in apoptotic effect in HEKa cells exposed to BCM over PCM or the control conditions.

Microarray analysis was performed on RNA gathered after 2 hours exposed to BCM, PCM, or the media control the samples of HEKa cells exposed for 0 hours were used as a control. The data were then filtered in order to identify differentially expressed genes that were associated with inflammatory responses, apoptosis and nitric oxide production in HEKa cells. There was an increase in transcriptional activity in HEKa cells exposed to BCM for genes associated with inflammation, apoptosis and NO production (Figure 2). In HEKa cells exposed to BCM some of the largest upregulation of transcriptional activity came from the genes CXCL2, IL-8, DUSP1, and ATF3. The DUSP1 gene is important for the regulation of p38 activation of LPS-activated macrophages. The p38 pathway plays a central role in multiple pathways associated with inflammatory response in many cell types [39,40]. The p38 MAPK pathway has been found to play a role in the production of inflammatory cytokines namely IL-1 and TNF-α but has also been found to contribute to the production of IL-8 in response to IL-1 osmotic shock and IL-6 in response to the production of TNF-α [41]. NFKBIA like DUSP1 is an important transcriptional regulator that induces innate and adaptive immune responses. NFKBIA is one of the genes that assist in the regulation of the magnitude and duration of inflammatory responses. One of its key roles is to prevent the inflammatory response from destroying excessive amounts of tissue [41]. Some of the other genes up regulated are associated with the production of inflammatory cytokines or chemokines. CXCL2, IL-6 and IL-8 are well known cytokines or chemokines which play important roles in mediating inflammatory responses to pathogen. CXCL2 and IL-8 are pro-inflammatory chemokines that assist in the mediation of neutrophil migration as well as the migration of other cellular and humeral factor components to the site of an infection. TNF-α, IL-6 and IL-8 were also upregulated but the fold change in expression over the control was much lower and the increase in expression was only seen in HEKa cells exposed to BCM. TNF-α, IL-6 and IL-1 are all multifunctional cytokines with a wide variety of functions. These three cytokines are interrelated with IL-1 and TNF-α inducing IL-6 and IL-6 in turn playing a role in the regulation of TNF-α [42]. TNF-α plays a role in tissue repair, inflammation and regulation of apoptosis as well as the activation of transcriptional factors such as NFΚB that are important to several processes including growth, death, and inflammation and stress responses [43]. While NF-κB is not regulated at the RNA level and is not be expected to be differentially expressed, Ingenuity Pathways Analysis software predicted its increased activation based on the expression patterns of 28 genes in the microarray results from BCM-treated but not PCM-treated HEKa cells relative to media controls (p = 1.06 x 10^-11^). DUSP1 is produced in human skin cells and specifies a protein with structural features similar to members of non-receptor-type protein-tyrosine phosphatase family which inactivates MAPKs. MAPKs play an important role in the human cellular response to environmental stress as well in the negative regulation of cellular proliferation [44]. This can lead to an increase in cell death and thus contribute to *S. aureus* biofilms’ negative effects on the ability of HEKa cells ability to respond to bacterial challenges and prevent apoptosis and promote reepithelialization.

Seven cytokines or chemokines commonly produced by keratinocytes in response to pathogens were tested using sandwich ELISAs [45-47]. After 4 hours, cells exposed to BCM showed statistically significant increases in concentrations of IL-6, TNF-α, and CXCL2 in HEKa cells exposed to BCM over HEKa cells exposed to PCM and the control media. The increase in inflammatory cytokine and chemokine responses after 4 hours of exposure to PCM, BCM, and the media control were in general agreement with earlier research by Secor et al. [6] who showed increases in inflammatory responses by HaCaT cells exposed to *S. aureus* biofilm products. HEKa cells exposed to PCM, BCM and the control media were also tested by ELISA’s for the same seven cytokines and chemokines. After 8 hours of exposure, levels of IL-8, along with previously elevated IL-6, TNF- α, and CXCL2 significantly increased in HEKa cells exposed to BCM over HEKa cells exposed to PCM and the media control. At the 8 hour time point HEKa cells treated with BCM showed a statistically significant increase in cytokines versus HEKa cells treated with PCM. These findings support the current hypothesis that biofilm-secreted products differ and have more dramatic effects than secreted products from planktonically grown bacteria which may contribute to difference between chronic and acute wounds [5]. The creation of biofilms enables bacteria to increase their interaction with one another and thus resist antibacterial and environmental pressures [6]. These biofilms are not only a congregate of bacterial cells but are also held together by a polysaccharide matrix. Understanding the components of the exopolysaccharide matrix produced by bacterial biofilms along with the difference in morphology of the cells may provide clues to the mechanisms of biofilms and why they produce the differential inflammatory responses that we have observed.

A somewhat unexpected finding from the microarray analysis was the upregulation of genes associated with nitric oxide production in HEKa cells exposed to BCM. Nine genes were up regulated in HEKa that were not found in control samples. Eight of those genes were unique to HEKa cells exposed to BCM and were not found in HEKa cells exposed to planktonically secreted products. Despite the genes associated with nitric oxide production there was not a significant upregulation of iNOS or NOS2 which are most often associated with nitrite production in cells. The lack of significant iNOS an NOS2 upregulation may have been due to the short time between the exposure of the HEKa cells to the PCM and BCM and the time of mRNA collections. Schnorr et al. reported that iNOS expression in keratinocytes was not detected until 4–8 hours after exposure to inflammatory cytokines and maximal expression does not occur until 24 hours post exposure [48]. Nitric oxide plays a key role in acute wound repair. Nitric oxide synthesis increases wound healing by enhancing collagen deposition within the wound and in dermal fibroblasts to enhance the mechanical strength of the tissue [29,30]. However, high concentrations NO may actually inhibit healing [31]. In this study, we observed upregulation of genes associated with NO production in cells treated with BCM, and tested the downstream effects of such genes by performing nitrite assays on culture supernatants. These nitrite assays were done in order to measure nitrite as an index of NO formation. Statistically significant increases in the amount of nitrite produced by HEKa cells exposed to BCM compared to HEKa cells exposed to PCM or just Epilife media were detected. These results add to the understanding of NO role in chronic wounds as opposed to acute wounds. The role of NO in biofilm production and dispersal is controversial. Falsetta et al. [49] found that in terms of *Neisseria gonorrhea* biofilms there was a high level of NO which seemed to aid the biofilm’s growth. Other studies have found that NO has been linked to the dispersal of *Pseudomonas aeruginosa* biofilms as well the prevention of biofilm formation in *S.aureus* and *Escherichia coli*[50,51]. It has been suggested that the disparate results are due to concentration of NO that is induced in the system. NO in large concentrations can be toxic to eukaryotic cells and in fact eukaryotic cells have developed defenses like metallothionein in response to oxidative stress [32]. Our results show that NO production by HEKa cells increased over time (Figure 4) as inflammatory effects of biofilm secreted products increased (Figure 3). Overproduction of reactive nitrogen species can impair cellular migration, proliferation, and synthesis of extracellular matrix that is important to wound healing by keratinocytes [35]. This lack of agreement between studies coupled with our results makes NO and its role in biofilm formation, dispersal, and/or chronic wound pathogenesis a topic that requires further investigation.

The majority of studies on immune responses to bacteria have been carried out with planktonically growing bacteria or cell components from planktonically growing bacteria. Infections due to *S. aureus* are characterized by strong inflammatory responses [52,53]. Several molecules such as Panton-Valentine leukocidin [54] sphingomyelinase [55], peptidoglycan, lipoteichoic acid [56,57], superantigens [58] as well as others have been shown to play a role in the inflammatory response directed towards *S. aureus*. It is possible that some of these same molecules may be responsible for the differences in inflammatory and nitric oxide responses we have measured; however, these molecules have not been specifically linked to *S. aureus* biofilms and chronic wound inflammation.

## Conclusion

Studies presented here further our understanding of the role that *S. aureus* biofilms may play in chronic wound pathogenesis. We found greater transcriptional activity in HEKa cells exposed to BCM compared to HEKa cells exposed to PCM or the media controls. The increased transcriptional activity was corroborated by increases in inflammatory cytokine production, nitric oxide production, and reduced cell viability. The increase in nitrite production in HEKa cells exposed to BCM is an intriguing finding that could be an important biofilm-mediated factor that contributes to the failure of chronic wounds to heal and warrants further investigation. Experiments to further understand the role of nitric oxide in chronic wounds and studies to identify *S. aureus* biofilm secreted factors responsible for the increased inflammatory responses are planned.

## Competing interests

We, the authors declare that we have no competing interests.

## Authors’ contributions

AT participated in the design of experiments, carried out experiments for cell viability, cytokine and nitric oxide detection, and Transcriptional profiling, participated in the statistical analysis of the data, and drafted the manuscript. MBF participated in the design of the transcriptional profiling experiments, directed microarray analysis, and help draft the manuscript. MB performed microarray analysis. RB conceived the study and participated in its design and coordination, participated in the data analysis, and helped draft the manuscript. All authors read and approved the final manuscript.
